# Recent Progress in the Membrane Distillation and Impact of Track-Etched Membranes

**DOI:** 10.3390/polym13152520

**Published:** 2021-07-30

**Authors:** Arman B. Yeszhanov, Ilya V. Korolkov, Saule S. Dosmagambetova, Maxim V. Zdorovets, Olgun Güven

**Affiliations:** 1 The Institute of Nuclear Physics, Ibragimov Str. 1, Almaty 050032, Kazakhstan; arman_e7@mail.ru (A.B.Y.); i.korolkov@inp.kz (I.V.K.); Dosmagambetova_s_s@mail.com (S.S.D.); 2 L.N. Gumilyov Eurasian National University, Satbaev Str. 5, Nur-Sultan 010008, Kazakhstan; 3 Ural Federal University, Mira Str. 19, Ekaterinburg 620002, Russia; 4 Department of Chemistry, Hacettepe University, Ankara 06800, Turkey

**Keywords:** track-etched membranes, membrane distillation, desalination, graft polymerization, hydrophobic membrane

## Abstract

Membrane distillation (MD) is a rapidly developing field of research and finds applications in desalination of water, purification from nonvolatile substances, and concentration of various solutions. This review presents data from recent studies on the MD process, MD configuration, the type of membranes and membrane hydrophobization. Particular importance has been placed on the methods of hydrophobization and the use of track-etched membranes (TeMs) in the MD process. Hydrophobic TeMs based on poly(ethylene terephthalate) (PET), poly(vinylidene fluoride) (PVDF) and polycarbonate (PC) have been applied in the purification of water from salts and pesticides, as well as in the concentration of low-level liquid radioactive waste (LLLRW). Such membranes are characterized by a narrow pore size distribution, precise values of the number of pores per unit area and narrow thickness. These properties of membranes allow them to be used for more accurate water purification and as model membranes used to test theoretical models (for instance LEP prediction).

## 1. Introduction

According to the World Health Organization, 1 billion people currently do not have access to clean tap water, and this number may increase to 3.5 billion by 2025 [1,2,3,4,5,6]. Among the new technologies for purifying drinking water, membrane processes are the most efficient and energy-conserving. Membrane water purification processes include microfiltration [7], ultrafiltration [8], nanofiltration [9], direct and reverse osmosis [10] and membrane distillation (MD) [11].

MD is emerging as one of the promising membrane technologies for the purification of waste and drinking water from various salts of heavy metals [12] and radionuclides [13] and in applications in food and textile industries [14,15] and pharmaceutics [16]. A typical MD process is composed of three stages: evaporation of the feed solution from the hot side of the membrane, transfer of vapor through the pores of the hydrophobic membrane and condensation of vapor on the permeate side of the membrane. In MD process, the porous membrane must be hydrophobic, allowing the passing through of only water vapor molecules but not bulk water. The membrane should also have good thermal stability to withstand high temperatures and low thermal conductivity to prevent heat loss across the membrane. Compared to other membrane separation methods, MD has significant advantages, such as high degree of purification from metal salts (more than 90%) and other nonvolatile compounds, relatively low operating temperatures and pressures and simplicity of hardware design.

The term “membrane distillation” was first introduced by Bodell in a patent [17], and in 1967, Findley [18] published the first scientific article on membrane distillation. He tested various types of membrane materials (aluminum foil, cellulose film, glass) in direct contact membrane distillation (DCMD). Silicone and Teflon have been used as materials to increase the hydrophobicity of the membrane. On the basis of these results, the requirements for the membranes to be used in MD were established. The scientific interest in the MD process, however, quickly faded away in the 1980s. The decline in popularity was due to several reasons such as the lack of suitable membranes for the process, the high cost of the membrane module and the poor water flux compared to reverse osmosis. With the development of new types of membranes and improved membrane modules, interest in MD began to increase again.

**Figure 1:**
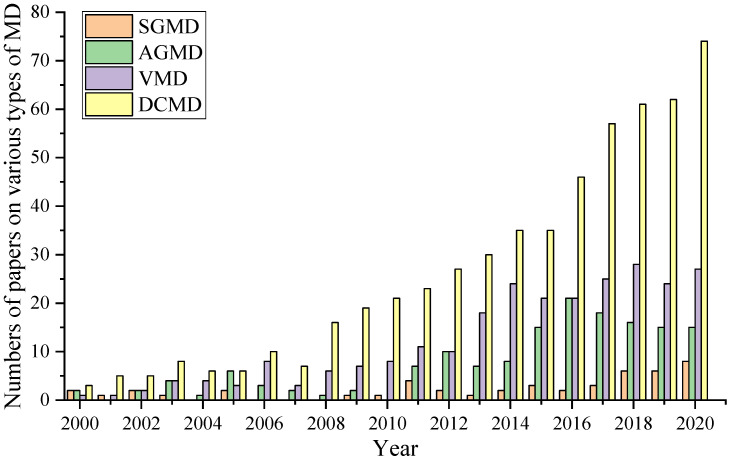
Number of publications on “membrane distillation” (searched with keywords “membrane distillation”, “DCMD” (direct contact membrane distillation), “VMD” (vacuum membrane distillation), “AGMD” (air gap membrane distillation), “SGMD” (sweeping gas membrane distillation)) from 2000 to 2020 (data taken from Science Direct database).

Over the past 20 years, the number of publications on the subject of “membrane distillation” has been growing every year, as is illustrated in Figure 1 where the numbers of publications on four types of membrane distillation configurations are shown.

Due to increasing number of publications on this subject, there has been a need for systematization and analysis of published works. There are a number of published review articles on this topic in the literature. Alkhudhiri et al. [11] considered mechanisms of heat and mass transfer, types of MD configurations and membrane characteristics. The review showed the necessity to collect data from large-scale MD studies, develop membranes with high hydrophobic character and further investigate the effect of operating parameters.

Nanofiber-based membranes prepared by electrospinning for use in MD were described in another review [19]. Membranes made from electrospun fibers showed high values for salt rejection and water flux. However, authors mentioned that finding the appropriate membrane structure, design and fabrication method are important to improve the performance.

Recent advances in electrospun nonfibrous membranes for MD were reviewed by Pan et al. [20], where both the original nanofiber membranes and its modification (for instance, with inorganic nanoparticles and fluorine-containing agents) were considered. Modification of membranes for MD led to enhancement of performance and degree of salt rejection. However, the authors refer to the problem of large-scale production of modified electrospun membranes and their fouling behavior.

Ghaffour et al. [21] presented new MD hybrids with energy efficiency enhancement. Perspectives of MD hybrid systems and the pros and cons of each hybrid process are discussed. Other authors [22] reviewed the possibility of using hybrid MD: hybrid MD bioreactor (MDBR) and MD forward osmosis (MD-FO) are especially successful combinations for wastewater treatment. MD pressure-retarded osmosis (MD-PRO) and MD reverse electrodialysis (MD-RED) also can be used for simultaneous recovery of water and energy. Ray et al. [23] evaluated the incorporation of new-generation nanomaterials such quantum dots, metal–organic frameworks (MOFs), metalloids and metal oxide based nanoparticles in MD membranes. The recent progress in photothermal membrane distillation (PMD) was reviewed by Razaqpur et al. [24], who illustrated new PMD mechanisms and membrane requirements. Fabrication and multistep modification of PMD membranes were also considered.

Achievements in improving the properties of membranes intended for MD have been presented in reviews [25,26]. The basic principles, configurations and required characteristics for membranes in MD were discussed to achieve the best results in water flux and salt rejection. Particular attention was paid to the production of hydrophobic membranes by phase inversion with different additives for pore formation, perfluorinated polymers and inorganic nanoparticles.

Advances in membrane modification to obtain omniphobic or Janus surfaces (conventionally, one side/interface of the membrane is lyophilic, whereas its other side/interface is lyophobic) allowed expanding MD for water purification from oil, surfactants and surfactant-stabilized emulsions [27,28].

Most of the reviews indicate that the limitations of membranes for large-scale MD consist in fouling, relatively high thermal conductivity of most popular hydrophobic polymers used in MD (PVDF, PTFE and PP) in comparison with PET or PS, high energy cost, expansion of production and improvement of water fluxes and purification degree.

In this review, we concentrate on using track-etched membranes (TeMs) in MD, which has not been discussed previously in review articles. Recent advances in main aspects of MD such as membrane distillation configurations, requirements for membranes and types of membranes are also elaborated. TeMs are characterized by regular pore geometry with the ability to control them per unit area, a narrow pore size distribution, a narrow thickness and a tortuosity of 1. Thus, such membranes have potential to be used as model membranes for development and confirmation of theoretical mass, heat transfer, LEP and fouling. Servi et al. [29] quantified the effects of initiated chemical vapor deposition polymer coatings on PC TeM permeability and LEP. Chamani et al. [30] developed a CFD-based genetic programming model for LEP estimation and tested it on TeMs. Furthermore, as mentioned above, the unique properties of TeMs can increase the accuracy of water purification.

## 2. Membrane Distillation Configurations

**Figure 2:**
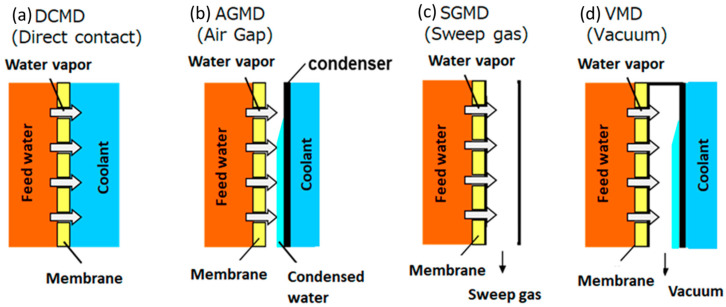
Scheme of typical MD configurations: (**a**) DCMD; (**b**) AGMD; (**c**) SGMD; (**d**) VMD [38].

As shown in Figure 2, there are four known and well-studied membrane distillation configurations: DCMD, AGMD, SGMD and VMD [31,32,33,34]. There are also known three supplemental configurations: liquid or water gap membrane distillation (LGMD/WGMD), thermostatic sweeping gas membrane distillation (TSGMD) and vacuum-assisted air gap membrane distillation (VA-AGMD) [35,36,37].

Direct contact membrane distillation (DCMD) is the simplest configuration in which the flow of hot and cold liquids is separated by a hydrophobic membrane. The pressure difference caused by the temperature gradient between the two sides of the membrane entails mass transfer through the pores of the membranes. In the MD process, only water vapor molecules penetrate through the pores of the membrane, while liquid molecules should not pass through the pores of the hydrophobic membrane. Due to the difference in vapor pressure, volatile compounds evaporate, while vapor molecules passing through the pores condense on the cold side (permeate). This configuration of MD is widely used in practice [39,40,41,42,43,44,45]; it is also well represented in Figure 1.

The configuration of AGMD assumes the presence of an air gap located between the cold side and the surface of the condensing liquid [46,47,48,49]. The vapor, passing through the membrane pores and air gaps, condenses on the surface of the cooling plate. In this configuration, the air gap is usually the determining factor in mass and heat transfer, which helps to increase the thermal efficiency of the process [50,51]. The air gap will reduce the heat loss by conduction through the membrane and decrease the efficiency of process. At the same time, the vapor should cross the air barrier, so the flux is reduced depending on the effective thickness of the air gap. The thickness of the air gap is an important factor that influences the AGMD performance [52]. The water flux increases with the decrease in the thickness of the air gap. This is probably due to the growing gradient of temperature inside vapor space [45,53]. It has been stated that when the thickness of the air gap is less than 5 mm, it significantly affects the membrane distillation process [54,55]. Pangarkar et al. [53] tested the effect of air gap thickness on the permeate flux using hydrophobic PTFE membrane in AGMD. The air gap thickness was varied from 1.2 to 3.2 mm. Results showed that permeate flux was reduced with increasing air gap thickness.

In SGMD, a sweeping gas is used on the permeate side as a carrier to remove vapor or collect vapor from the membrane surface [56,57,58]. As with air gap membrane distillation, the flow of gas reduces heat loss and significantly increases mass transfer. However, the use of this type of configuration is inappropriate due to poor water flux and the necessity of large volumes of sweeping gas, which entails additional costs [59].

Vacuum membrane distillation (VMD) is based on the application of vacuum from the permeate side. In this configuration, the surface on which the vapor condenses is separated from the membrane by creating a vacuum on the other side of the membrane. The disadvantages of this configuration are the difficulty in hardware design associated with the installation of expensive condensers and large pressure drops on the membrane surface that can lead to a decrease in hydrophobicity [60,61,62,63,64,65].

In liquid or water gap membrane distillation (LGMD or WGMD), the gap between the membrane and the plate is filled with distilled water. Water evaporating from the hot side diffuses through the pores of the membrane and condenses in the liquid gap. Large heat losses make this method less practical to use [35,66,67,68].

In a thermostatic sweeping gas membrane distillation (TSGMD), an inert gas is passed between the membrane and the condensation surface (cold wall). The presence of a condensation surface on the cold side lowers the temperature of the sweeping gas, which leads to an increase in driving force and cleaning efficiency. However, this configuration is currently not promising since there are difficulties in hardware design [36].

Thus, as can be seen from the published articles, DCMD is the most well studied and efficient configuration in MD technology. DCMD is characterized by low energy consumption and simple hardware design, and the process is carried out at relatively low temperatures. Among the shortcomings, it is worth noting the impossibility of using MD in the separation of highly volatile mixtures [69,70,71].

## 3. Requirements for Membranes to Be Used in MD

The main characteristic of a membrane to be used in MD is high hydrophobicity to keep the liquid phase from penetrating through the pores of the membrane. In practice, membranes made of polypropylene (PP), poly(vinylidene fluoride) (PVDF) and poly(tetrafluoroethylene) (PTFE) perfectly suit to this requirement. Moreover, the membranes must have liquid entry pressure (LEP) of at least 2.5 bar. LEP is the pressure required for fluid to flow through the pores of the membrane. Usually, membranes with pore diameters from 0.1 to 1 μm are used in MD, as a further increase in the pore diameter negatively affects the LEP value [72].

It has been argued that water flux and mass transfer are reduced with increasing membrane thickness, while small thickness leads to heat losses that negatively affect the driving force of the process [73,74]. Therefore, an optimal membrane thickness [75] has been considered to be from 10 to 400 μm for various applications. Porosity is another important factor for membranes, with a high value leading to an increase in water flux. Typically for membranes, the porosity varies from 30 to 90%, including for membranes obtained by the electrospinning method [76].

Thus, the membrane to be used in MD should have the following properties:

1. LEP value of at least 2.5 bar.
2. Narrow pore size distribution to reduce the risk of pore wetting.
3. The recommended pore size of membranes is from 0.1 to 1 μm.
4. The optimum membrane thickness should be between 10 and 60 µm. Thicker membranes (>60 μm) should be used in the purification of highly concentrated mixtures.
5. The porosity of the membrane should be as high as possible.
6. The contact angle of membranes must be as high as possible (>90°).

## 4. Membranes for MD

### 4.1. Type of Membranes

#### 4.1.1. Flat-Sheet Membranes

Various types of membranes are used in the MD process. In the separation and purification technologies over the past 50 years, flat membranes have been the most studied and widely used [77]. This type of membrane is suitable for all types of MD configurations such as DCMD, AGMD, and VMD. The main advantages of flat membranes are ease of manufacturing, assembly, operation and testing, making them most suitable for use in the membrane applications.

#### 4.1.2. Spiral-Wound Membranes

Spiral-wound membranes in the MD were investigated for the desalination of brackish water and seawater [78,79,80,81,82]. The following polymers were used as membrane materials: PP, poly(vinyl chloride) (PVC), polyethylene (PE), PTFE and synthetic resins [83]. The structure of rolled membranes includes the material itself, a mesh pad, a permeate carrier and a membrane support layer that forms a cap which is wrapped and twisted around a perforated tube for collecting permeate. However, spiral-wound membranes have not been preferred in MD because of significant drawbacks (difficulty in cleaning and replacing contaminated areas) [78,84].

#### 4.1.3. Tubular Membranes

In addition to flat-sheet and spiral-wound membranes, tubular membranes have been studied for the desalination of marine, brackish, and waste water. Tubular membranes have been used in DCMD, VMD and AGMD [85,86,87,88]. Among the main advantages, it is worth noting a low tendency towards contamination, ease of maintenance and a good contact area, making them attractive for commercial applications. However, with the development of MD, the use of tubular membranes decreased due to the advent of more efficient flat-sheet and hollow fiber membranes.

#### 4.1.4. Hollow Fiber Membranes

In recent years, hollow fiber membranes have become the most commonly used membranes in MD process. According to Camacho [43], the materials used for the manufacture of hollow fiber membranes are mainly made of PP, PVDF and PVDF–PTFE. The hollow fiber membrane module has the highest component density [11,89], the best effective surface area per unit volume and high efficiency [77]. In addition, hollow fiber membranes can operate at very high pressures (above 100 bar) [44] and consume much less energy. Despite this, hollow fiber membranes have significant disadvantages. Wang and Chung [90] determined that the main disadvantages of this type of membrane are poor water flux and inferior mechanical properties. In addition, hollow fibers tend to become dirty [91], and replacing damaged fibers is a time-consuming and costly process. Besides MD, hollow fiber membranes are used in liquid extraction, desalination and wastewater treatment.

As a result, various types of membranes are used in the MD process, regardless of the type of the process itself. Among the promising types of membranes, hollow fiber membranes are worth noting; however, the inconvenience in operation and the weak mechanical properties are significant disadvantages. The most optimal types of membranes used in MD are flat-sheet types.

### 4.2. MD Membrane Fabrication Techniques

Membranes for MD can be prepared by stretching, phase inversion and electrospinning processes [25,92,93,94]. Several types of membranes have been prepared using a combination of different methods. Zhu [95] developed a new hydrophobic hollow fiber PTFE membrane by cold pressing, including extrusion, stretching and sintering.

Stretching is a solvent-free method in which membranes are made by extruding a polymer at a temperature close to its melting point to form micropores [96]. This method of fabrication is cheaper than other techniques. In stretching a polymer with a partial crystallinity stretched to the axis of crystallite orientation, the polymer is extruded at temperature below its melting point to produce a film. With this technique, membranes with high porosity (90%) and uniform porous structure can be produced [97,98].

Phase inversion is a phase separation process involving the controlled transfer of a polymer from a solution to a solid state. Fabrication by phase inversion can be divided into the following steps: first, polymer pellets are dissolved in a solvent to form a casting solution, which is then cast on a plate. Then, the semiliquid film is cast on the plate and immersed into the bath for precipitation. Finally, a polymeric film is formed with an asymmetric or symmetric structure [97].

This method can be used to prepare both asymmetric and symmetric porous membranes using various methods, namely nonsolvent-induced phase separation (NIPS), thermally induced phase separation (TIPS), vapor-induced phase separation (VIPS) and evaporation-induced phase separation (EIPS). The first two methods are most commonly used for manufacturing hydrophobic membranes [99].

The preparation of hydrophobic PVDF membranes by the NIPS method is discussed in [100]. Hydrophobic membranes with a high contact angle (~148°) were obtained with low surface energy, without surface modification. First, the nascent membrane was immersed into alcohol solution and then immersed in water coagulation bath for precipitation of polymer. Modified membranes were tested in DCMD, and the water flux and salt rejection were found to be close to those of commercially available PVDF membranes [101].

The electrospinning technology was proposed for the manufacture of nanofiber membranes for MD [19,20,76,93]. Electrospinning is an effective method of fabricating nanofibrous membranes with high porosity and roughness. This technique consists of three major components: a high-voltage electric source, a syringe with a metallic needle and a collecting roller. A high voltage is used to create an electrically charged jet of the polymer solution. Polymer membranes obtained by electrospinning have a high surface area to pore volume ratio [102]. Electrospun membranes have been prepared from various polymers, including PVDF [103,104,105], PVDF–SiO2 [106], polystyrene (PS) [107], PTFE–polyvinyl acetate [108] and PVDF–HFP/SiNP [109]. It should be noted that electrospun membranes, as some of the most popular membranes, have been tested in all types of configurations.

Kebria [104] and others proposed a method for increasing the hydrophobicity of nanofiber PVDF membranes by introducing dendritic structures during electrospinning. Dendritic structures were synthesized by polycondensation between the hydroxyl groups of boehmite and the carboxyl groups of nitriloacetic acid. The effect of different amounts of dendritic structures on membrane morphology, elemental composition and surface hydrophobicity was assessed by SEM and contact angle measurements. The contact angle increased from about 129 to 139°, and water flux and salt rejection were 11 kg/m^2^·h and 99%, respectively.

Ke et al. [107] managed to prepare hydrophobic PS membranes by electrospinning with a fiber diameter ranging from 150 to 240 nm by varying the polystyrene concentration from 8 to 12%. Sodium dodecyl sulfate was used as an additive. The modified nanofiber membrane had a fairly high porosity (more than 80%), a narrow pore size distribution, and a high contact angle (more than 100°). Hydrophobic membranes were tested in desalination of sea water by membrane distillation for 10 h of operation with the water flux of 31 kg/m^2^·h. Feng and colleagues [110] were the first to use nanofiber PVDF membranes in the MD process in the purification of NaCl saline solutions. The water flux and salt rejection were 11,000–12,000 g/m^2^·h and 99%, respectively. Prince et al. [111] succeeded in increasing the contact angle of PVDF membranes obtained by electrospinning from 80° to 154° by embedding hydrophobic clay nanoparticles in the polymer mat. Hydrophobic membranes showed good salt rejection from 98.2 to 99.9%.

Duong et al. [112] investigated the preparation of nanofiber styrene–butadiene–styrene membranes (SBS) by electrospinning. Compared to commercially available PTFE membranes, SBS membranes were found to have higher contact angle and salt rejection values. Among the disadvantages of SBS membranes, poor water flux compared to PTFE can be mentioned. Khayet et al. [113] studied two-layer nanofiber membranes with different hydrophobic properties (PVDF–polysulfone) prepared by electrospinning. It was found that the two-layer type of membranes exhibits better water flux in desalination compared to single-layer membranes. The water fluxes of two-layer nanofiber membranes at salt concentrations of 12 and 30 g/L were ~50,000 and ~48,000 g/m^2^·h, and salt rejection was 99%.

It has been concluded that electrospinning is a reliable way to obtain hydrophobic membranes [26,102,114]. Relatively low cost, variability in the use of various polymers and materials and the possibility of obtaining fibers with diameters from several nanometers to several microns make this method of fabrication more effective in comparison with the others [115].

### 4.3. Main Membrane Materials

Currently, there are various polymeric membrane materials such as PTFE, PP, PVDF [116,117,118] and poly(ethylene terephthalate) (PET) used in MD.

Among the above-listed materials, PTFE ((-C_2_F_4_-)_n_) has the lowest surface energy of about 9–20·10^−3^ N/m [119]. It is a highly crystalline polymer with excellent thermal and chemical resistance. PTFE membrane is often manufactured by sintering or melt extrusion [120,121].

PP ((-CH_2_-CH[CH_3_-])_n_) is also a highly crystalline thermoplastic but has a higher surface energy (30·10^−3^ N/m) than PTFE [43]. Porous PP membranes are generally manufactured by the melt extrusion method [122,123,124], as well as by thermally induced phase separation [87,125]. Compared to other membranes used in MD, PP is relatively advantageous in terms of materials and production costs. However, poor water flux and moderate thermal stability at elevated temperatures complicate its use in MD [126].

PVDF ((-CF_2_-CH_2_-)_n_) has almost the same surface energy (~30·10^−3^ N/m) as polypropylene. Unlike the other polymers used in MD applications, PVDF can be easily dissolved in some solvents such as n-methyl-2-pyrrolidone, dimethylacetamide and dimethylformamide [127,128,129], and it can also be melt-processed easily due to its low melting point of 170 °C.

Recently, PET ((-C_10_H_8_O_4_-)_n_) membranes have begun to be investigated in the process of MD [130,131,132,133]. PET is a polymer with high chemical and heat resistance and good resistance to organic solvents and acids, and it has a relatively low thermal conductivity in comparison with PTFE. However, the main disadvantage of PET for MD is its semi-hydrophobic properties (CA 55–83°) [134]. In order to use PET membranes in MD, it is necessary to significantly increase its hydrophobic properties. Improvement of hydrophobicity can be achieved by, for example, modifying the surface via application of a hydrophobic coating or grafting various hydrophobic groups onto the surface, as will be elaborated further in this paper.

### 4.4. Membrane Modification Methods

Hydrophobicity is the main property of a membrane that plays an important role in improving the water flux. Basically, membranes with a high contact angle (more than 90°) are used; however, there are some membranes with less hydrophobic surfaces. Various modification methods such as the addition of pore-forming agents [135], perfluorinated polymers and inorganic nanoparticles [136] were used to improve the hydrophobic properties and increase the water flux.

Simone et al. [135] modified a microporous hydrophobic fibrous PVDF membrane. Poly(vinylpyrrolidone) was used as a pore-forming agent. Hydrophobic membranes were tested in vacuum membrane distillation, with distilled water as the feed. The water flux varied from 3.5 to 18 kg/m^2^·h at 50 °C at a vacuum pressure of 20 mbar. The disadvantages of this method are the insufficient water flux in salt purification and the necessity to maintain a vacuum.

Edwie et al. [136] developed a method for the preparation of hydrophilic–hydrophobic bilayer PVDF hollow fiber membranes using a hydrophobic modifier, silica. The samples were tested in DCMD of sodium chloride solution with methanol. The maximum water flux reached 84 kg/m^2^·h, and the salt rejection was above 90%. However, the stability of the two-layer hydrophilic–hydrophobic membrane was low.

García-Payo et al. [137] modified poly(ethersulfone) (PES) membranes using a solution of trimethylsilyl chloride and trimethylmethoxysilane by the sol–gel method. The modification of the membrane surface led to an increase in the contact angle to 119°. Hydrophobic membranes were tested in the MD process. The water flux and salt rejection were 4.47 kg/m^2^·h and 99%, respectively. The main disadvantage of this method was the nonuniformity of the hydrophobic coating.

Efome [138] prepared PVDF–SiO_2_ hydrophobic membranes by the phase inversion method. The obtained membranes were characterized by scanning electron microscopy, measurement of contact angle, and infrared (IR) spectroscopy. The salt rejection was above 98% with a salt concentration of 35 g/L.

Generally, the most effective modification methods are membrane surface modifications, which allow one to widely change the characteristics of membranes, such as roughness, hydrophobicity and surface energy. In addition, hydrophilic polymers can be hydrophobized in this way.

Coating the membrane surface is the simplest way to instantly improve the hydrophobicity of membranes by applying a thin functional layer to the surface. Surface coating is often carried out by sol–gel [139], spinning, immersion [140] and other methods [141]. Graft polymerization is considered one of the effective methods for improving membrane hydrophobicity. The membrane surface can be modified by the formation of covalent bonds between the membrane and the grafted chains. Unlike the coating process, graft polymerization improves the chemical stability of the graft layer. In other words, it can completely solve the problem of hydrophobic layer instability. Surface grafting can be achieved by using various methods, such as plasma- and radiation-induced graft polymerization [116] and photo- and thermal-initiated graft polymerization of a single monomer or a mixture of two or more monomers [116].

Plasma treatment is the process of adsorption and polymerization of ionized gas onto the membrane surface. Modification of the polymer surface is carried out under the influence of high-energy ions, reactive species and photons generated during the discharge. The effectiveness of plasma treatment depends on various parameters such as type of plasma (DC, radiofrequency, microwave), discharge power density, pressure and flow rate of the gas mixture in the chamber and treatment time. Plasma treatment of polymers in inert gases (for instance, He and Ar) is effective for creating free radicals and does not add new chemical functional groups from the gas phase. Plasma treatment in H_2_O or O_2_ atmosphere is used to create polar functional groups that can significantly increase the free energy of the polymer surface. The main advantage of this method is a uniform surface treatment is achieved and the depth of the modified layer is several nanometers.

During plasma treatment, the monomer is pumped into a vacuum chamber. Further, under the action of the lamp, the monomer is ionized with the formation of reactive species to generate free and active radicals. These radicals are adsorbed and subsequently polymerize on the membrane surface, creating a dense coating layer [142].

Wei et al. [143] proposed a method of plasma treatment of hydrophilic poly(ethersulfone) (PES) membranes under carbon tetrafluoride (CF_4_) atmosphere. The results showed that the modification resulted in a significant increase in the contact angle above 120°. The water flux of permeate reached 43 kg/m^2^·h with a salt rejection of more than 98%.

Plasma modification of a PAN membrane by carbon tetrafluoride (CF_4_) was also applied in VMD [144]. The authors proposed a method for purifying ethyl acetate from an aqueous solution by vacuum membrane distillation. The influence of plasma modification conditions, surface porosity and separation efficiency of modified membranes were investigated. It was found that the contact angle of PAN membrane increased from 42 to 124°, but the water flux was within 8 kg/m^2^·h.

Tooma [145] studied the modification of PVC with ethyl acrylate by radiation-induced graft polymerization. The success of grafting ethyl acrylate onto the PVC surface was confirmed by IR and energy dispersive X-ray (EDX) spectroscopy. The water flux of permeate was found to increase about 15 times. The maximum water flux was about 37 kg/m^2^·h at a concentrate temperature of 60 °C and a vacuum pressure of 2 mbar.

Liu et al. [146] prepared a hydrophobic membrane based on PES by radiation graft polymerization of 1H,1H,2H,2H-perfluorodecyl methacrylate. After grafting the monomer, the contact angle was found to increase to 114°, and the pore diameter of the membrane slightly decreased. Membrane distillation was carried out in vacuum mode; the water flux was 50 kg/m^2^·h, and the salt rejection was more than 90%. Among the disadvantages, the short operating time of the membrane may be noted.

UV graft polymerization has been a frequently used grafting method recently. Salehi et al. [147] developed a method for chemical modification of PP flat sheet membrane surface by UV graft polymerization of acrylic acid, 2-hydroxyethyl methacrylate and ethylene glycol dimethacrylate. Hydrophobic membranes were tested in membrane distillation; the water flux at different ratios of monomers varied from 3 to 8 kg/m^2^·h, and the salt rejection was more than 98%.

A brief summary of results for the modification of various types of membranes is presented in Table 1.

Thus, it can be concluded that there are many modification methods, such as graft polymerization (photo- and thermal-initiated, plasma, radiation), the sol–gel method and the method of coating the membrane surface. Modification of membranes can significantly increase and diversify the properties of the material. The choice of the modification method depends on the convenience, simplicity of hardware design and cost-effectiveness.

### 4.5. Fouling Phenomena

Membrane fouling is the process of precipitation of solutions or particles on the surface or in the pores of a polymer that causes a decrease in permeate flux. Although membrane fouling is one of the key problems in membrane distillation, it is still poorly understood. Typically, there are several types of fouling in membrane distillation: biological, inorganic and organic fouling [148,149].

#### 4.5.1. Biological Fouling (Biofouling)

The presence of bacteria or microorganisms on the membrane surface leads to the appearance of biofouling. However, biofouling in MD occurs extremely rarely in comparison with other types of fouling. Since the feed solution has a high salinity and the MD process occurs at relatively high temperatures, the probability of the survival of bacteria is extremely small. This type of fouling is less studied in MD [150,151]. Krivorot et al. [152] studied biofouling behavior using hydrophobic PP membranes in the MD process. Results showed that there was a small drop in water flux when operating at 40 °C with cross-flow DCMD. Under these conditions, SEM images show the presence of microorganisms on the membrane surface. An increase in temperature from 40 to 70 °C led to a decrease in biofouling on the membrane surface. Nthunya et al. [153] researched the influence of silver nanoparticles (AgNPs) on the biofouling in MD. Modified nanofiber PVDF–AgNPs might be a good choice for the mitigation of biofouling.

#### 4.5.2. Inorganic Fouling

Inorganic fouling occurs quite frequently in the MD process and is one of the key problems in water desalination. Inorganic fouling in MD forms due to precipitation and crystallization of salts present in feed solutions. In the MD process, the main scales are sodium chloride (NaCl), sulfates of basic metals (CaSO_4_, BaSO_4_, MgSO_4_), phosphates and calcium carbonates, aluminum and iron oxides, etc. Gryta et al. [154] studied the effect of salt concentration (NaCl solution) on the permeate flux in MD. Authors observed that there is a significant decrease in permeate flux at concentrations up to ~48.9 g/L. The effects of calcium carbonate in the feed solution on the permeate flux were studied by Qin et al. [119]. Decomposition of calcium carbonate in VMD and SGMD was faster than in DCMD. This is related to the solubility of CaCO_3_, which is inversely proportional to temperature. Therefore, the high temperature of the feed solution promotes CaCO_3_ crystal formation.

#### 4.5.3. Organic Fouling

Deposition of organic matter such as polysaccharides, proteins, carboxylic acid and humic acid on the membrane surface leads to the appearance of organic fouling in MD [155]. Organic fouling in DCMD was investigated by Naidu et al. [156]. They studied organic fouling with the use of model solutions of humic acid (HA), polysaccharide and bovine serum albumin (BSA). Results showed that polysaccharide demonstrated minimal fouling due its hydrophobic nature, whereas BSA and HA on the membrane surface significantly reduced permeate flux by 50%. Khayet et al. [157] used HA solutions with various concentrations by DCMD using PTFE and PVDF membranes. The obtained results showed that permeate flux decreased by 8% after 30 h of work.

## 5. Track-Etched Membranes in Membrane Distillation

**Figure 3:**
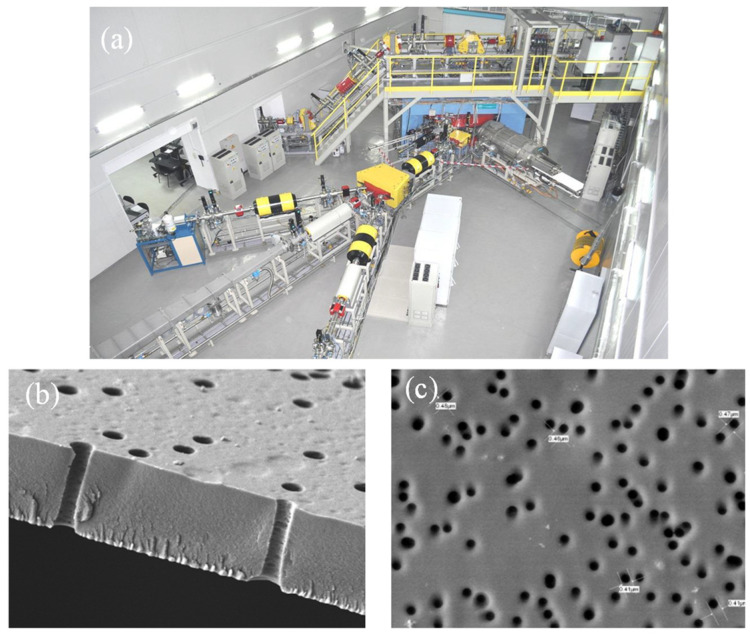
DC-60 cyclotron complex (**a**); SEM image of cross-sectional view of the membrane (**b**); SEM image of PET TeM surface (**c**).

Track-etched membranes (TeMs) are prepared by irradiation of polymer thin films with swift heavy ions and subsequent photosensitization and chemical etching for pore size control [158,159,160,161,162,163,164,165]. A top view of the DC-60 cyclotron complex at the Nuclear Physics Institute in Nur-Sultan, Kazakhstan, and typical SEM images of cross-section and the top-surface view of PET TeMs are shown in Figure 3.

This technique is known for its ability to accurately control narrow pore distribution and pore size. The size and density of pores can be adjusted in a wide range: from a few nanometers to 15 μm for size and from 10^6^ to 10^9^ pores/cm^2^ for density. The porosity of the membrane is mainly determined by the duration of irradiation and the pore size, and pore geometry is mainly determined by the etching time, temperature, concentration, additives and etching bath configuration [166,167,168,169,170]. It should be noted that the mechanical properties of PET film may change during TeM fabrication. With an increase in membrane porosity, mechanical properties of the membrane decrease. For instance, for PET TeMs with a pore size of 400 nm and pore density of 4 × 10^7^, the burst strength is 304.1 ± 7.0 kPa [171], which is quite high for practical use. The chemical composition of the surface of PET also changes during the etching process. In [164], it was shown that the concentration of carboxyl groups increases from 0.84 nmol/cm^2^ for pristine PET to 6 nmol/cm^2^ after photosensitization and chemical etching.

Manufacturing of TeMs in Kazakhstan has been performed in the laboratories of the Institute of Nuclear Physics in Nur-Sultan in the DC-60 accelerator complex (Figure 3a). This complex is composed of three channels, one of which is being used for irradiation of polymers for TeM production.

A characteristic feature and advantage of TeMs is the regular geometry of pores with the ability to control their number per unit area and narrow distribution of pore sizes. This, in turn, provides the target selectivity and water flux of membranes [172]. TeMs are widely used, for example, in the processes of precision ultrafiltration and microfiltration of liquids and gases; in the analytical control of substances; and in food and pharmaceutical industries, microelectronics and other areas of science and industry [173,174,175].

PC, PVDF and PET are the most widely used polymer films (5–24 µm) for the production of TeMs. These polymer films have differences in contact angle, thermal conductivity and cost. PVDF film is more expensive in comparison with PET and PC; moreover, etching PVDF membranes is more complex and requires high temperature (150 °C) and pressure (4 atm), which makes it difficult to use them in large-scale production.

PET has the lowest thermal conductivity (0.15 W/mK, whereas that of PC is 0.2 W/mK and that of PVDF is 0.19 W/mK) [176]. The lower the thermal conductivity, the more energy-efficient the MD process will be.

PVDF is a more hydrophobic polymer (90°) in comparison with PET (73°) and PC (82°). However, after channel formation, contact angle of PET TeMs varies from 40 to 55° [164], depending on pore size, while that of PC TeMs is ~55° [177] and that of PVDF TeMs is ~49–72° [158]. Thus, all films need to be hydrophobized to meet the requirements for membranes in MD.

Laricheva et al. [158] used unmodified PVDF TeMs with CA of 49–72° in AGMD of salt solutions. They achieved a water flux of 38,100 g/h·m^2^ with salt retention of 99%. The authors explain this by the fact that the observed contact angle of the surface does not correspond to reality, but represents the apparent contact angle itself. The porosity of the solid surface of the membrane reduces the apparent contact angle. However, in this work, there are not enough data to conclude whether such a hydrophilic membrane can be used in MD. The authors did not present LEP analysis, which can show the applicability of the membrane for MD and the possible leakage of water. Moreover, slat rejection was calculated only by changes in the conductivity of feed solution; permeate solution was not analyzed, which can lead to overestimated results in terms of the degree of salt rejection.

Methods of PET TeMs hydrophobization for MD were developed by the authors of [133] and our group [130,131,178,179]. PET has high strength and chemical and heat resistance. It is resistant to various low-concentrated or nonconcentrated acids and alkalis and is practically insoluble in most organic solvents. Moreover, it has low cost and low thermal conductivity properties. Thus, PET is an attractive membrane material for the TeMs to be used in MD.

The effective use of TeMs in MD and also in the processes of direct osmosis and filtration requires the expansion of the range of their characteristics (pore size and structure, hydrophobicity/hydrophilicity, the creation of special chemical groups on the surface). The development of methods for the intended modification of PET TeMs while preserving the pore structure to achieve specific physicochemical properties and performance characteristics is a challenging technological task [29,158]. Below, we consider the current methods of hydrophobization of TeMs for use in MD.

### 5.1. Hydrophobization of PET Track-Etched Membranes by Covalent Bonding of Silanes

**Figure 4:**
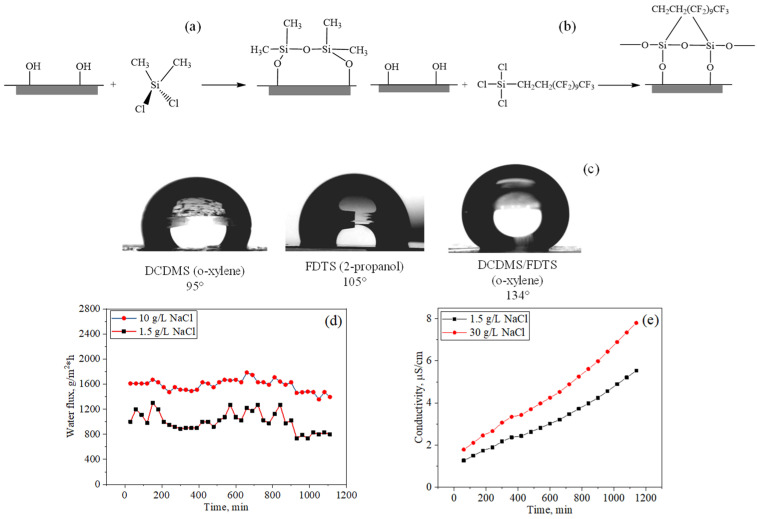
Scheme of modification of PET TeMs by covalent bonding of DCDMS (**a**) and FDTS (**b**); contact angle of modified PET TeMs (**c**); water flux (**d**) and conductivity (**e**) for NaCl solutions with concentrations of 1.5 and 10 g/L. Adapted from [178].

Hydrophobization of PET TeMs by covalent bonding of silanes is a simple and effective way to change the hydrophilic–hydrophobic properties of the surface without altering the pore structure by creating a thin layer of a hydrophobic agent. Scheme of PET TeMs modification by covalent bonding shown in Figure 4.

The polycondensation reaction between the surface of PET TeMs and 1H,1H,2H,2H-perfluorododecyltrichlorosilane (FDTS) or dichlorodimethylsilane (DCDMS) proceeds due to the high reactivity of the Si–Cl bond, which is sensitive to hydrolysis and interaction with hydroxyl groups on the PET TeMs surface. The etching process introduces numerous hydroxyl and carboxyl groups on the etched surface of PET.

Various reaction parameters affecting the grafting degree and the value of the contact angle have been studied. The morphology of the membrane surface also depends on the modification conditions. With an increase in concentration and time, an increase in roughness occurs. It was found that effective grafting with preservation of the pore structure was achieved by using 20 mM FDTS solution in 2-propanol and a reaction time of 24 h. In this case, the water contact angle was 109°. The obtained hydrophobic membranes were tested in the purification of a saline solution of NaCl with a concentration of 1.5–30 g/L. The average water fluxes were 1005 and 97 g/m^2^·h at concentrations of 1.5 and 30 g/L, and the salt rejection was 99.5% and 98.4%, respectively. A significant decrease in water flux has been observed with increasing salt concentration, which is probably due to the gradual contamination of the membrane surface [178].

### 5.2. Hydrophobization of PET Track-Etched Membranes by Photo-Initiated Graft Polymerization

Photo-initiated graft polymerization is characterized by the fact that it does not significantly affect the substrate by changing its properties since the radiation energy is low and the grafting takes place under mild conditions [180]. Moreover, during graft polymerization, stable covalent bonds are formed with the surface, which brings stability to the hydrophobic layer. Thus, it appears as a convenient method suitable for hydrophobization of membranes for use in MD. Graft polymerization usually takes place in two stages: immobilization of the photoinitiator/photosensitizer at the inner walls of the nanochannels of the membrane and then graft polymerization of the monomers from the membrane surface. Results of some recent studies on graft modification of PET TeMs for MD applications from this laboratory are briefly described in the following section.

#### 5.2.1. Photo-Initiated Graft Polymerization of Triethoxyvinylsilane (TEVS)

**Figure 5:**
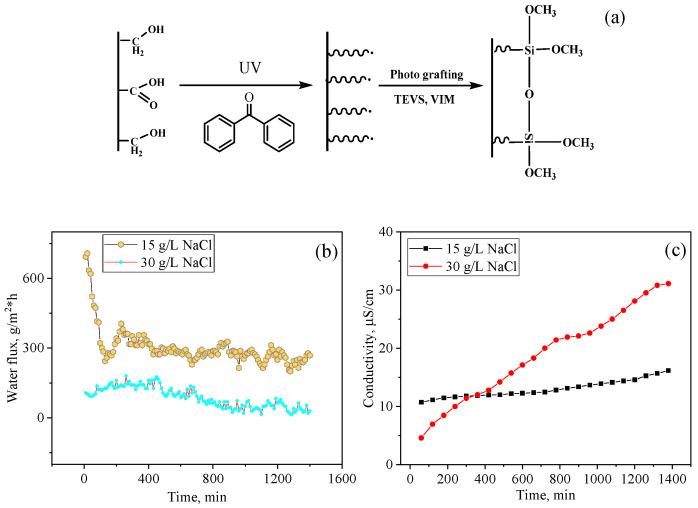
Scheme of surface modification of PET TeMs with TEVS (**a**); water flux (**b**) and electrical conductivity (**c**) for NaCl solutions with concentrations of 15 and 30 g/L. Adapted from [130].

As shown in Figure 5, photo-initiated graft polymerization was carried out in several stages: the photosensitizer benzophenone (BP) was first adsorbed onto the membrane surface from a 5% dimethylformamide (DMF) solution. BP-immobilized PET TeMs were then soaked in TEVS solution and UV irradiated. The degree of grafting was found to be very low as this monomer has a low tendency toward graft polymerization [181].

In order to increase the degree of grafting, a monomer (N-vinyl imidazole) with a high tendency towards graft polymerization was introduced into TEVS solution in amounts from 0.3 to 6.6%. Various parameters (time, concentration of monomers and additives) influencing the degree of grafting were studied, as the degree of grafting significantly affects the membrane morphology and pore size. A high degree of grafting may lead to complete clogging of pores in the polymer. In radiation-induced grafting of acrylic acid inside the nanochannels of PVDF TeMs, it was observed that the pores were completely filled after 40% of grafting [182].

The use of VIM together with TEVS made it possible to obtain membranes with a high CA value of 104.9°. The LEP value for modified membranes was >0.43 MPa, which makes them applicable for MD. The prepared samples were tested in the purification of 15 and 30 g/L NaCl saline solution by the MD method. The average water fluxes were 295 and 88 g/m^2^·h for solutions with concentrations of 15 and 30 g/L with purification degrees of 99.3% and 95.2%, respectively [130].

#### 5.2.2. Photo-Initiated Graft Polymerization of Styrene

**Figure 6:**
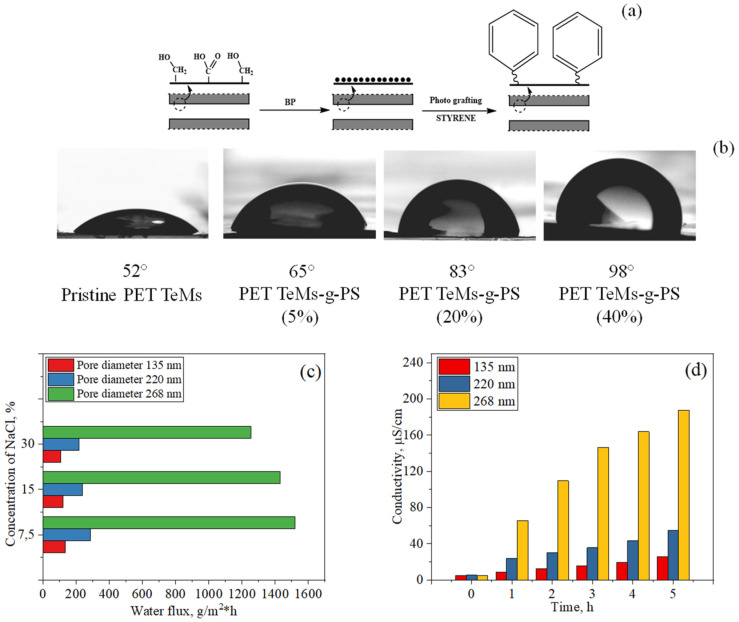
Scheme of surface modification of PET TeMs by styrene grafting (**a**); contact angle of the pristine and modified PET TeMs (**b**); water flux (**c**) and electrical conductivity (**d**) of NaCl solution with a concentration of 15 g/L. Adapted from [179].

Scheme of modification PET TeMs by styrene grafting is shown in Figure 6. The method of modification by graft polymerization of styrene on the surface of PET TeMs was also studied in [179]. Various parameters influencing the degree of grafting were studied. After the graft modification, the surface morphology was found to become smoother than that of the pristine PET TeMs. A slight decrease in the pore diameter was observed, which is expected due to the formation of a polystyrene layer inside the channels. With an increase in the concentration of styrene, the value of the CA was found to increase significantly; at a concentration of 40%, the value reached was 98°. Analysis of previously published works on using styrene as a hydrophobic agent shows us that the contact angle of different materials can be increased up to 94–104° [158,159,160]. Thus, full coverage of PET TeMs was achieved. From the AFM images presented in [179], it can be seen that roughness increased from 2.15 ± 0.04 nm to 5.16 ± 1.01 nm with grafting. Thus, the hydrophobicity of the membrane is almost totally due to the grafting of hydrophobic polystyrene, and full coverage of PET TeMs was achieved.

Hydrophobized membranes with different pore diameters were used in the purification of saline solution with a concentration of 7.5–30 g/L by MD. The average water fluxes were 286, 238, and 219.3 g/m^2^·h for solutions with concentrations of 7.5, 15 and 30 g/L, respectively, for membranes with pore diameters of 220 nm, and the salt rejection varied from 97.5 to 98.9% [179].

### 5.3. Hydrophobization of PET Track-Etched Membranes by Immobilization of Silica Nanoparticles

**Figure 7:**
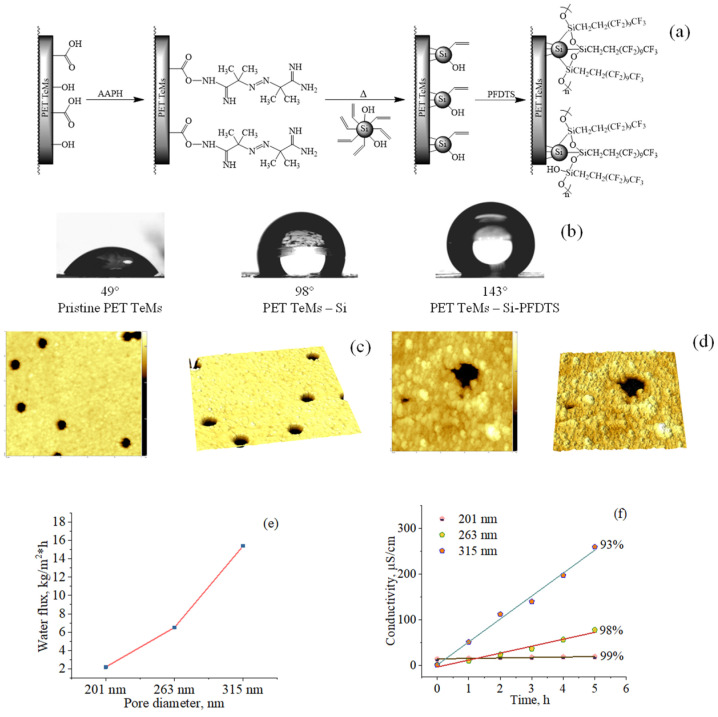
Scheme of modification by immobilization of silica nanoparticles (**a**); CA of the pristine and modified PET TeMs (**b**); AFM surface images of pristine PET TeMs (**c**) and modified PET TeMs with Si NPs (size 5 × 5 μm) (**d**); the water flux (**e**) and electrical conductivity (**f**) of NaCl solution with a concentration of 30 g/L with different pore diameters. Adapted from [131].

Another strategy to introduce hydrophobic properties of PET TeMs is simultaneously applying hydrophobic agents together with increasing the roughness. For this purpose, the possibility of immobilization of silica nanoparticles (Si NPs) on the membrane surface was studied. Hydrophobization of PET TeMs by immobilization of silica nanoparticles is shown in Figure 7. First of all, Si NPs with C=C bonds were synthesized from TEVS by the sol–gel method [183]. The resulting solution of silica nanoparticles in ethanol was subsequently used to modify PET ion-track membranes. The modification of PET ion-track membranes was performed according to the scheme presented in Figure 7.

In the first stage, 2,2′-azobis(2-methylpropionamidine) hydrochloride (AAPH) was covalently bonded with COOH end-groups of PET via activation with pentafluorophenol and N-(3-dimethylaminopropyl)-N’-ethylcarbodiimide (EDC).

In the second stage, the ethanol solution of prepared Si NPs was passed through the membranes from both sides, using a vacuum pump to fill the nanochannels with Si NPs. Membranes with Si NPs were immersed in an ethanol solution of 0.2% AAPH. The solution was purged with argon and kept at 75 °C for 3 h to initiate immobilization of Si NPs on the surface of PET ion-track membranes. After the reaction, membranes were washed in ethanol to flush away loose NPs and dried.

In the third stage, prepared membranes were modified with 1H,1H,2H,2H-perfluorodecyl triethoxysilane (PFDTS). Hydrophobization led to a significant increase in the CA up to 143°. The morphology of the membrane was investigated by AFM, and it was found that the roughness of modified membranes increased from 3.7 to 15.5 nm. Testing of hydrophobic membranes in the purification of saline NaCl by the MD showed the water flux of ~15 kg/m^2^·h, and salt rejection reached 99%.

Thus, an increase in roughness together with the immobilization of hydrophobic chemical groups made it possible to obtain hydrophobic membranes with high water flux [131].

### 5.4. Hydrophobization of PET Track-Etched Membranes by Plasma Deposition of Fluoropolymers

Another method of PET TeMs surface hydrophobization is the plasma deposition of fluoropolymers [133]. Due to their unique properties, fluoropolymers are well suited for plasma surface polymerization. This method has several advantages, such as high deposition degree and relatively safe and simple process. The modification was carried out on PET TeMs using various plasma parameters (reaction time, distance between electrodes, pressure). The deposition degree was proportional to the plasma treatment time. The values of the CA varied in the range of 85–95° ± 3. The hydrophobic membranes were used in concentrating apple juice by MD. At the same time, the membrane modified with perfluorohexane showed a higher value of water flux (~2850 mL/m^2^·h) than commercial analogs made from PTFE for fruit juice solution (~2100 mL/m^2^·h). The degree of sugar removal was 98–100%.

### 5.5. Application of Hydrophobized PET TeMs in Water Contaminated with Pesticides

**Figure 8:**
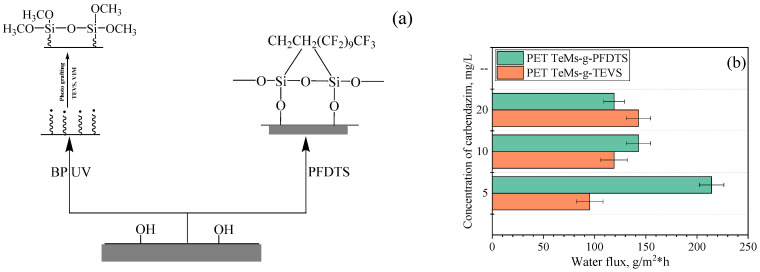
Scheme of surface modification of PET TeMs (**a**); water flux for modified PET TeMs-PDFTS and PET TeMs-g-TEVS (**b**) of pesticide solution (carbendazim) with different concentrations. Adapted from [184].

Hydrophobization of PET TeMs described above was carried out in two ways: by UV-induced graft polymerization of TEVS and covalent binding of PFDTS. Scheme of modification and water flux for mod189ified PET TeMs are shown in Figure 8.

Hydrophobized PET TeMs membranes were tested to decontaminate water from pesticides (carbendazim) with concentrations of 5, 10 and 20 mg/L. Table 2 shows the contact angle and pore size values before and after modification. The average water fluxes at these concentrations of carbendazim for PET TeMs-PFDTS were 214, 142.85 and 119 g/m^2^·h, respectively. The average water fluxes for PET TeMs-g-TEVS were 95.2, 119.2 and 142 g/m^2^·h for the above-listed concentrations. It was found that the concentration of carbendazim measured by UV spectroscopy in all selected samples was below the detection limit (100 μg/L) [184].

### 5.6. Application of Hydrophobized PET TeMs in Liquid Low-Level Radioactive Waste Treatment

MD is an effective method for water treatment of liquid low-level radioactive waste (LLLRW). In our previous work [179], LLLRW samples were taken from the secondary circuit of the WWR-K research reactor (Almaty, Kazakhstan, Institute of Nuclear Physics) and concentrated by the MD method using modified PET TeMs-g-PS. The efficiency of salt rejection was monitored by atomic emission spectroscopy for the analysis of the main ions in LLLRW, such as Na, Mg, K, Fe, Ca, Al, Sb, Sr, Mo and Cs (Table 3). The gamma spectrometer was used to monitor the activity of some radioisotopes: ^60^Co, ^137^Cs and ^241^Am. In the experiment, membranes with different pore diameters (142, 206, and 242 nm) were tested. According to Table 3, all degrees of rejection were higher than 90%, most of them being close to 100%.

**Figure 9:**
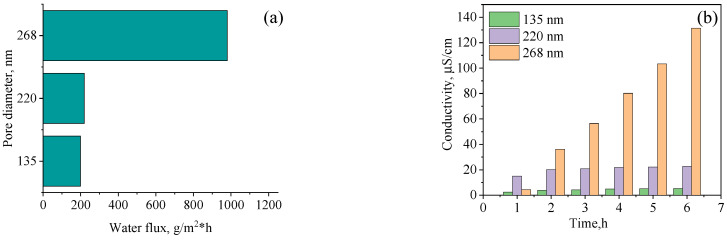
Water flux (**a**) and electrical conductivity (**b**) during continuous DCMD tests using hydrophobized PET TeMs-g-PS with different pores for radioactive waste solution. Adapted from [179].

Results of water flux and electrical conductivity during DCMD are shown in Figure 9. The average water fluxes for membranes with pore diameters of ~135 and 268 nm were 198.5 and 980 g/m^2^·h, respectively.

Results on decontamination factors of radioisotopes are presented in Table 4. PET TeMs-g-PS with pore diameters of 220 nm showed decontamination factors of >85 for ^60^Co, >1727 for ^137^Cs and 5 for ^241^Am. It should be noted that in most cases the results obtained were below detection limits.

LLLRW was also purified with MD by using other types of membranes (see Table 5). However, it is difficult to compare various results with each other, since the source of contaminated waters was different, and therefore the composition of LLLRW was also different. However, our group made a comparison between TeMs and nanofiber PTFE [179], and relevant results are presented in Table 3 and Table 4. The decontamination factor of PTFE membrane is 10 for ^60^Co, 439 for ^137^Cs and >2 for ^241^Am; the degree of purification is several times lower than that of TeMs.

The results of the modification of PET TeMs for use in membrane distillation are summarized in Table 6.

It was observed that the immobilization of silica nanoparticles leads to the highest hydrophobization of the TeM surface, which makes it possible to modify membranes with large pore diameters (up to 350 nm), which allows achieving water flux of 15 kg/m^2^·h. The main challenge of membrane hydrophobization is to find methods that allow the hydrophobization of membranes with the largest pore diameter, which in turn will lead to high porosity and water flux while maintaining a high degree of purification. As can be seen from Table 6, covalent bonding of FDTS, photo-initiated graft polymerization of TEVS, photo-initiated graft polymerization of styrene, immobilization of Si nanoparticles and plasma deposition of fluoropolymers led to sufficient hydrophobization of TeMs with pore diameters of 220, 200, 220, 315 and 400 nm, respectively. Thus, the last two methods have prospects for further use, since modified membranes have high values of performance and purification degree, and they can compete with other types of membranes (see Table 1). It should be noted that the main disadvantage of TeMs is their low porosity, which limits water fluxes. However, as shown in [179], TeMs with narrow pore size distribution led to better water purification from salts and LLLRW in comparison with nanofiber PTFE membranes.

There is also the problem of evaluation of the degree of salt rejection. This parameter has been estimated in different ways by different authors. Some of them take into account changes in conductivity only in the feed solution, while others use the following equation: R% = 1–TDS_permeate_/TDS_feed_. This equation gives an overestimated value of the degree of salt rejection, since it does not take into account the volume of liquid from the permeate side, and evaporation of water from the hot side is also possible.

In our works, for a more accurate evaluation of the degree of salt rejection (R), the use of the following equations is proposed [131]:

(1) $$R\;=\;100\;-\;(\frac{C_{\mathrm{real}}}{C_{\mathrm{fic}}}\;\times \;100\%)$$

(2) $$C_{\mathrm{real}}\;=\;\frac{\Delta \sigma \;\times \;1000}{2.3}$$

(3) $$C_{\mathrm{fic}}\;=\;\frac{\Delta m\;\times \;C_{\mathrm{feed}}}{m_{p}}$$

where R—degree of salt rejection,%; C_real_—concentration of NaCl in permeate side after MD, g/L, calculated according to conductivity (conductivity of 1 mg/L NaCl solution is 2.3 µS/cm); C_fic_—theoretical concentration of NaCl (provided that feed solution passed without purification), g/L; Δσ—difference in conductivity of permeate solution before and after MD, µS/cm; 2.3 mS/cm—change in the conductivity of the solution with the addition of 1 g/L of NaCl; Δm—permeate gain after MD, g; C_feed_—initial concentration of salt in feed solution, g/L; m_p_—mass of water from the permeate side before MD, g.

Thus, different methods for estimation of the degree of salt rejection do not allow direct and reliable comparison of the data obtained. Therefore, it is necessary to adopt and use a universally agreed method of calculation of salt retention.

## 6. Conclusions

The studies presented in this review allow us to say that TeMs have the prospect of being used in membrane distillation. Specific features of TeMs such as controlled pore size and narrow pore size distribution and thickness lead to more efficient water purification, and this is clearly seen in the purification of low-level liquid radioactive waste. On the other hand, the low porosity of such membranes limits their water flux; nevertheless, water fluxes have an average value in comparison with nanofiber or electrospun membranes. Thus, we are confident that TeMs can be used in the precision treatment of hazardous wastes. Moreover, further developments in the modification of track-etched membranes for membrane distillation may result in the formation of omniphobic and Janus surfaces with the aim to expand the use of such membranes in the separation of oil- and surfactant-containing aqueous systems.

Moreover, we would like to emphasize that such membranes can be obtained with precisely defined channel sizes and porosity; that is, the number of these channels per cm^2^ can be controlled with high accuracy. Thus, such membranes have the potential to be used as model membranes for the development and confirmation of models, including for the MD process. For instance, they have been used to confirm a model for LEP prediction. We believe that by grafting biocidal polymers and polymers showing affinity to specific compounds and metals, research in this field will widen the use of TeMs in MD applications.

## Figures and Tables

**Table 1:** Properties of some membranes used in MD modified by different methods.

Type of Membrane	Modification Method	Contact Angle, °	Feed Solution	Salt Rejection, %	Water Flux g/m^2^·h	Ref.
PVDF	NIPS	148	NaCl	99	87,400	[100]
Nanofiber PVDF	Electrospinning	139	NaCl	99.9	10,700	[104]
Nanofiber PS	Electrospinning	113	NaCl	99.9	31,000	[107]
Nanofiber PVDF	Electrospinning	154	NaCl	99	5800	[111]
Nanofiber SBS	Electrospinning	132	NaCl	99.9	10,500	[112]
PVDF–polysulfone	Electrospinning	130	NaCl	99.9	49,000	[113]
PES	Sol–gel	119	NaCl	99.3	44,700	[118]
Bilayer PVDF	Addition of perfluorinated polymers	~135	NaCl	99.9	83,400	[127]
PVDF–SiO_2_	Phase inversion	92	NaCl	99.9	2900	[138]
PES	Plasma treatment	120	NaCl	99.9	42,000	[143]
PVC	Radiation-induced graft polymerization	96	Water	/	37,500	[145]
PES	Radiation-induced graft polymerization	114	NaCl	99.98	50,500	[146]
PP	UV graft polymerization	138	NaCl	97	3000–8000	[147]

**Table 2:** Properties of PET TeMs before and after hydrophobization.

Sample	Contact Angle, ±4°	Effective Pore Size, nm	Pore Size (from SEM Analysis), nm	LEP, MPa
Initial PET TeMs	58	198 ± 5	220 ± 8	0.12
PET TeMs-g-TEVS	89	167 ± 8	216 ± 3	>0.43
PET TeMs—PFDTS	134	148 ± 6	174 ± 4	>0.43
Initial PET TeMs	55	302 ± 8	310 ± 15	0.015
PET TeMs-g-TEVS	85	287 ± 10	292 ± 20	0.04
PET TeMs—PFDTS	115	274 ± 12	285 ± 18	0.04

**Table 3:** Chemical composition of the LLLRW sample and the effluent after DCMD process.

Element	Concentration in the Feed (μg/L)	Concentration in the Permeate (PET TeMs-g-PS, d = 268 nm) (μg/L)	Concentration in the Permeate (PET TeMs-g-PS, d = 220 nm) (μg/L)	Concentration in the Permeate (PET TeMs-g-PS, d = 135 nm) (μg/L)	Concentration in the Permeate (PTFE Nanofiber Membrane d = 220 nm) (μg/L)
Cs (σ = ± 26%)	304	1.45	0.33	<0.05	34.3
Mo (σ = ± 15%)	458	1.11	<0.3	<0.3	76.0
Sr (σ = ± 15%)	136	<0.5	<0.5	<0.5	11.1
Sb (σ = ± 15%)	46.3	<0.3	<0.3	<0.3	8.96
Al (σ = ± 16%)	660	<3	<3	<3	<30
Ca (σ = ± 16%)	1780	55.3	52	44	208
Fe (σ = ± 10%)	383	<0.6	<0.6	<0.6	<6
K (σ = ± 15%)	249,200	377	414	150	7476
Mg (σ = ± 15%)	1046	2.52	4	2	<10
Na (σ = ± 15%)	4,710,000	13,200	3200	540	601

**Table 4:** Radioisotope composition of feed waste solution and permeate solution after DCMD process.

Radioisotope	Activity of the Feed (Bq/kg)	Activity of the Permeate (PET TeMs-g-PS, d = 220 nm) (Bq/kg)	Decontamination Factor (D)	Activity of the Permeate (PTFE Nanofiber Membrane d = 220 nm) (Bq/kg)	Decontamination Factor (D)
^60^Co	85.4 ± 6.1	<1.0	85	16.5 ± 1.1	10
^137^Cs	1900 ± 27	<1.1	1727	4.33	439
^241^Am	<2.2	<0.45	5	>0.49	2

**Table 5:** A comparative analysis of the water flux, salt rejection and decontamination factor for some isotopes for different types of membranes used in LLLRW applications.

Type of Membrane	Water Flux of LLLRW, g/m^2^·h	Salt Rejection, %	Decontamination Factor for Isotopes	Reference
Hydrophobic PET TeMs-g-PS	980	99.9	^60^Co—85 ^137^Cs—1727 ^241^Am—5	[179]
PTFE membrane	5000	90–95	^60^Co—10 ^137^Cs—439 ^241^Am >2	[179]
PTFE spiral-wound membrane	1300–1800	>93	^60^Co—4336.5 ^137^Cs—43.8	[13]
PES membrane	70,000–159,000	>90	^60^Co—400–1000 ^137^Cs—900–1400 ^85^Sr—400–800	[185]
PP hollow fiber membrane	6300	99.6	Co (simulated)	[186]
Ceramic NF membrane	20,000	99.9	Co (simulated)	[187]
Hydrophobic PP membrane	7100–30,300	/	^85^Sr—10^5^ ^60^Co—10^4^ ^137^Cs—10^3^	[188]
PP hollow fiber membrane	5000–50,000	>90	^85^Sr—3700 ^60^Co—8300 ^137^Cs—6000	[189]

**Table 6:** Dependence of the LEP values and the contact angle on the pore size of the PET TeMs modified with different methods and their performance in MD process.

Modification Method	Pore Size, nm	Contact Angle, °	Water Flux, g/m^2^·h	Salt Rejection, %	LEP, MPa	Reference
Covalent bonding of FDTS	410 ± 14	104	/	/	0.012	[178]
Covalent bonding of FDTS	305 ± 13	107	/	/	0.039	[178]
Covalent bonding of FDTS	220 ± 11	109	97—for 30 g/L NaCl	98.4	0.340	[178]
Photo-initiated graft polymerization of TEVS and VIM	200 ± 18	105	88—for 30 g/L NaCl	95.2	>0.430	[130]
Photo-initiated graft polymerization of styrene	268 ± 21	91	1254—for 30 g/L NaCl	83.2	0.140	[179]
Photo-initiated graft polymerization of styrene	220 ± 15	99	219.3—for 30 g/L NaCl	97.5	0.340	[179]
Photo-initiated graft polymerization of styrene	135 ± 15	104	107.7—for 30 g/L NaCl	98.1	0.390	[179]
Immobilization of silica nanoparticles	315 ± 6	125	15,000—for 30 g/L NaCl	93	0.350	[131]
Immobilization of silica nanoparticles	263 ± 5	132	6500—for 30 g/L NaCl	98	0.430	[131]
Immobilization of silica nanoparticles	201 ± 5	135	2200—for 30 g/L NaCl	99	>0.430	[131]
Plasma deposition of fluoropolymers	400	85–95	1100–2900	95–100	/	[133]

## Data Availability

The data presented in this study are available on request from the corresponding author.
